# Anki Use and Academic Performance in Medical Education: A Systematic Review of Evidence and Learning Theory

**DOI:** 10.1007/s40670-026-02643-5

**Published:** 2026-01-17

**Authors:** Nicholas Frappa, Danil Chernov, Morgan Dillon, Matthew G. Alben

**Affiliations:** 1 Jacobs School Of Medicine and Biomedical Sciences, 955 Main Street, Buffalo, NY 14203 USA; 2 Department of Orthopaedics and Sports Medicine, University at Buffalo, Buffalo, NY USA

**Keywords:** Anki, Medical students, Spaced repetition, Retrieval practice, Academic performance, Systematic review

## Abstract

**Background:**

Anki, an open-source flashcard platform that integrates spaced repetition and retrieval practice, has become a widely used study tool among medical students. Despite its popularity, evidence regarding its academic impact remains limited and heterogeneous.

**Methods:**

We conducted a systematic review of PubMed, CINAHL, ERIC, PsychINFO, Scopus, Embase, and Web of Science to identify studies evaluating Anki use and academic performance in undergraduate medical education. Inclusion criteria required measurement of exam outcomes in medical students using Anki. Eleven eligible studies were qualitatively synthesized.

**Results:**

Three studies demonstrated a consistent positive association between regular Anki use and USMLE Step 1 performance. High-frequency users outperformed minimal users by 4–13 points, with one study identifying a dose-response effect based on total cards reviewed. Evidence for university-administered exams was more mixed: some studies found significant benefits with structured Anki programs, while others reported no measurable difference despite positive student perceptions. These discrepancies may reflect differences in deck quality, usage consistency, and shorter preparation timelines for in-course exams. Only one study assessed Step 2 CK and found no significant benefit. Included studies varied considerably in methodology, definitions of Anki “use,” and outcome measures.

**Conclusion:**

Anki use is associated with higher performance on standardized examinations that emphasize foundational knowledge, including the USMLE Step 1. Findings for course-based examinations were mixed and may depend on contextual factors. Overall, the results are consistent with established learning theory, but evidence is largely observational. Future studies using longitudinal designs and objective measures of Anki use are needed to better define its educational impact.

**Supplementary Information:**

The online version contains supplementary material available at 10.1007/s40670-026-02643-5.

## Introduction

Medical education demands the acquisition and retention of an immense volume of knowledge, spanning basic sciences, clinical medicine, and procedural skills. This cognitive load is compounded by increasingly condensed curricula that demand rapid assimilation of content. Without effective strategies for retention, students may struggle to apply foundational knowledge in clinical contexts and perform suboptimally on high-stakes assessments such as the United States Medical Licensing Examination (USMLE) or institution-specific exams [1]. Prior research has shown that biomedical knowledge decays rapidly unless reinforced through active review, with significant attrition occurring within the first year of study [1, 2].

Over the past century, cognitive psychology has identified retrieval practice, defined as the active recall of information, and spaced repetition (the distribution of study sessions over time) as key strategies for optimizing long-term retention [3, 4]. The foundation for these methods can be traced to the seminal work of Hermann Ebbinghaus [5], who demonstrated that memory decays exponentially and that spaced review at increasing intervals can effectively combat this decline. Modern analyses have since validated Ebbinghaus’s forgetting curve, showing that memory loss is most pronounced shortly after initial learning [6]. Beyond the timing of review, Roediger and Karpicke [4] demonstrated that repeated retrieval, testing oneself rather than passively reviewing, enhances long-term retention more effectively than re-exposure. These principles are integral to medical training, where durable retention of foundational knowledge supports clinical reasoning and performance on high-stakes assessments [7].

In recent years, medical students have increasingly turned to Anki, a free, open-source flashcard application designed to support long-term retention through spaced repetition and active recall. Anki uses an algorithm originally based on SuperMemo 2 (SM-2), the second-generation spaced-repetition model developed for the SuperMemo program. A newer algorithm, the Free Spaced Repetition Scheduler (FSRS), has since been introduced to improve interval optimization based on user performance [8]. Unlike many digital study tools that rely on learner-directed review, Anki uses an adaptive spaced-repetition algorithm to determine which items are due for review on a given day, shifting review scheduling from the learner to the software. This design allows Anki to support durable retention of large volumes of factual information by minimizing forgetting through automated, performance-based spacing [8]. The platform reinforces retrieval-based learning by prompting users to generate answers before revealing them, thereby strengthening memory consolidation through active recall. Because Anki is designed to promote long-term recall of factual knowledge, its intended outcomes align closely with the performance measures most frequently examined in the literature—standardized and course-based examinations that emphasize accurate retrieval of declarative information [4, 7]. Associations between Anki use and test performance are therefore conceptually expected, as both rely on the strengthening of factual memory through effortful retrieval [9]. Additionally, Anki’s asynchronous, user-driven format may also foster self-regulated learning behaviors, including goal-setting, progress monitoring, and metacognitive reflection—skills that are critical in both undergraduate and lifelong medical learning. Collaborative platforms such as AnkiHub and pre-curated decks like AnKing have become popular resources for students seeking streamlined, high-yield content aligned with USMLE preparation [10–12]. In a national survey of 560 medical students from 102 U.S. medical schools, Halperin et al. [12] found that 68.3% of respondents reported using Anki, a rate that exceeded that of many traditional study tools. Another indication of Anki’s widespread adoption is the existence of large online communities dedicated to the platform. At the time of writing, the Reddit forum “r/medicalschoolanki” had over 175,000 members, exceeding the number of U.S. medical students and underscoring the tool’s central role in modern medical education [13].

Despite this enthusiasm, the empirical literature evaluating Anki’s impact on academic performance remains limited and heterogeneous. While some studies report modest positive associations between Anki use and USMLE Step 1 performance, such as gains of approximately one point for every 1,700 cards reviewed [14], others find no significant benefit in exam performance [15]. Most existing studies rely on self-reported usage patterns and are observational in nature, with varying definitions of what constitutes “Anki use.” For instance, as summarized in a scoping review by Barrison et al. [11], some studies define use as the mere presence of the app on a device, while others track total cards reviewed, deck types used (premade vs. user-generated), or consistency of daily engagement. While some studies, such as Wothe et al. [16], have explored outcomes beyond test scores (e.g., sleep quality, stress, extracurricular involvement), the majority of the literature remains narrowly focused on academic performance, with few examining broader constructs such as metacognition and perceived productivity [11]. Nonetheless, Anki’s widespread adoption, open-source flexibility, and algorithmic alignment with learning science have positioned it as the most representative example of digital retrieval-based study tools used by medical students.

To the best of our knowledge, this is the first systematic review to synthesize the available evidence examining the association between Anki use and academic performance in medical students. Given the theoretical foundation supporting retrieval-based learning and the platform’s widespread adoption across medical schools, a comprehensive synthesis is needed to guide students, educators, and researchers. Prior studies differ widely in methodology, outcomes assessed, and definitions of “use,” making it difficult to draw generalizable conclusions from individual studies. This systematic review aims to evaluate the relationship between Anki use and medical student examination performance, focusing on standardized exams such as the USMLE Step 1 and Step 2, the Comprehensive Osteopathic Medical Licensing Examination of the United States (COMLEX-USA) equivalents, and institutionally administered assessments. By identifying patterns and limitations across the existing literature, we hope to clarify the academic impact of Anki and inform future research on study strategies in medical education.

## Methods

### Literature Search Strategy

A systematic literature search was conducted using PubMed (MEDLINE) CINAHL, ERIC, PsychINFO, Scopus, Embase, and Web of Science databases to identify studies evaluating Anki and spaced repetition in undergraduate medical education. The search was performed on October 12th, 2025, using six distinct keyword combinations: “Anki” AND “medical student,” “Anki” AND “medical school,” “spaced repetition” AND “medical student,” “spaced repetition” AND “medical school,” “retrieval practice” AND “medical student,” and “retrieval practice” AND “medical school.” No date limits were applied. Filters were used to include only English-language studies involving human participants. Search terms were selected to maximize sensitivity for studies evaluating spaced repetition learning in medical education while maintaining specificity for Anki.

### Study Selection

All records identified through the search were imported into Covidence (Veritas Health Innovation, Melbourne, Australia) for screening. After automatic duplicate removal, titles and abstracts were independently screened by two reviewers. Full texts of potentially eligible articles were then reviewed in detail to determine final inclusion. Studies were included if they investigated Anki use among medical students and reported a measurable academic or testing outcome, such as performance on standardized exams or course assessments. We included studies referring to undergraduate medical students if participants were enrolled in a medical degree program, regardless of entry pathway or country-specific terminology. Studies were excluded if they (1) did not focus on medical students, (2) did not assess Anki specifically, (3) lacked measurable academic or testing outcomes, or (4) were not primary research (e.g., reviews, editorials). Discrepancies at any stage of screening or full-text review were resolved by discussion and consensus. These criteria were selected to ensure inclusion of primary studies directly evaluating Anki use among medical students with objective academic or testing outcomes. This systematic review was conducted in accordance with the Preferred Reporting Items for Systematic reviews and Meta-Analyses (PRISMA) 2020 guidelines for systematic reviews and meta-analyses [17]. The review protocol was not prospectively registered.

### Data Extraction and Synthesis

Data from each included study were extracted into a standardized spreadsheet. Extracted variables included first author, year, country, study design, sample size, type and frequency of Anki or spaced repetition use, outcome measures assessed, and key findings. Data extraction was performed independently by two reviewers, with discrepancies resolved through discussion and consensus. Due to substantial heterogeneity in study designs, definitions of Anki use, and outcome metrics, meta-analysis was not feasible. Instead, results were synthesized qualitatively following systematic review guidelines consistent with an integrative systematic review framework, which allows inclusion of diverse study designs and both quantitative and qualitative evidence through thematic analysis [18, 19]. This approach was appropriate given the aim to examine how Anki use relates to medical student learning and performance rather than to evaluate a single standardized intervention. Methodological quality of included studies was assessed using the Medical Education Research Study Quality Instrument (MERSQI) [20]. Item-level scores were assigned independently by two reviewers, with discrepancies resolved by consensus. Detailed scoring is provided in Supplementary Table 1.

## Results

### Literature Search

**Fig. 1:**
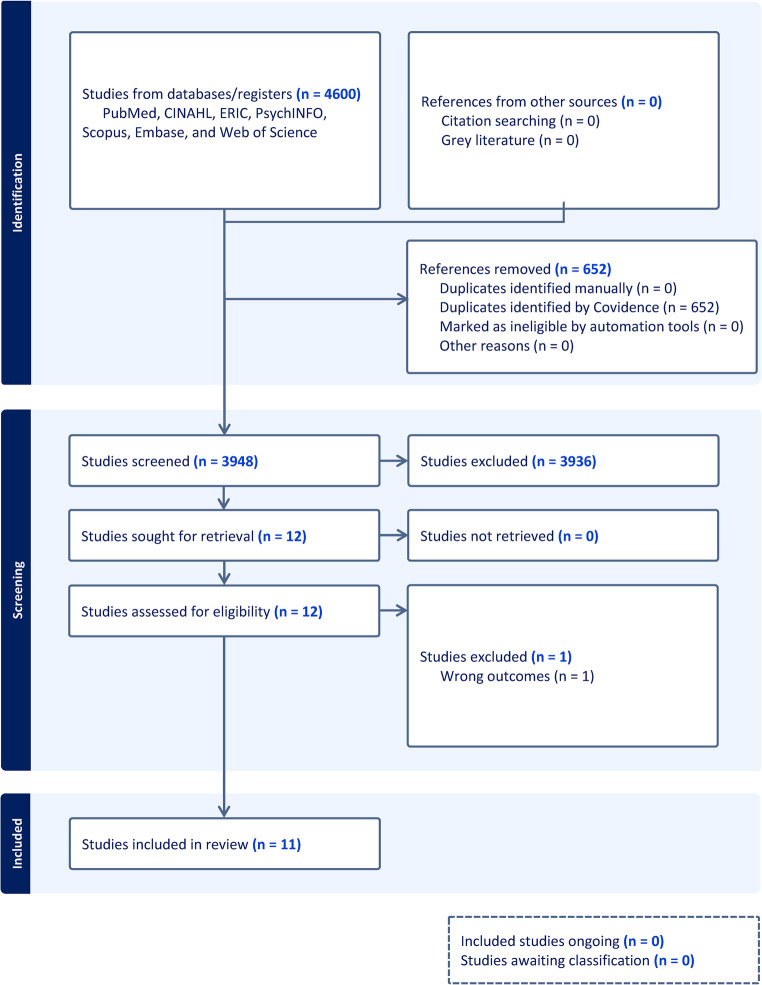
PRISMA flow diagram summarizing the study selection process

The database searches yielded 4600 records. After removal of 652 duplicates, 3948 titles and abstracts were screened. Of these, 12 full-text studies were assessed for eligibility, with 1 excluded for not reporting relevant academic outcomes. Eleven studies met all inclusion criteria and were included in the final review. The full selection process is illustrated in the PRISMA flow diagram (Fig. 1).

### Study Characteristics

Eleven studies met inclusion criteria, published between 2015 and 2025, encompassing a variety of designs, sample sizes, and methods for assessing Anki use. Nine studies were conducted in the United States, one in Ireland [21], and one in Pakistan [22]. Study designs included prospective cohort studies (*n* = 3), cross-sectional (*n* = 5), quasi-experimental studies with historical or parallel control groups (*n* = 2), and one mixed-methods prospective study [21]. Sample sizes ranged from 25 to 206 participants, with a combined total of 1,135 medical students across all studies.

Definitions of Anki use varied across studies. Five studies [21, 23–26] used objective tracking methods such as database file submission or scraper-based logging to quantify flashcard counts, review frequency, or study duration. Four studies [14, 16, 27, 28] relied on self-reported usage patterns, often dichotomized as regular versus minimal use or stratified by frequency and dependency. Two studies [15, 22] provided faculty-developed Anki flashcards without measuring individual usage. Outcome measures varied and included standardized exams such as USMLE Step 1 or 2 CK, Comprehensive Basic Science Examination (CBSE), and institution-specific course assessments. Study characteristics are summarized in Table 1.

**Table 1:** Characteristics of studies included in the systematic review

First Author (Year)	Study Design	Sample Size	Definition of Anki Use	Key Findings
Deng (2015)	Cross-sectional survey	72	Self-reported number of unique Anki flashcards reviewed during preclinical Step 1 preparation	Number of unique Anki flashcards was an independent predictor of USMLE Step 1 score (B = 5.9 × 10⁻⁴, *p* = 0.024). Each additional ~ 1700 Anki cards reviewed was associated with a 1-point increase in Step 1 score.
Durrani (2024)	Quasi-experimental study with parallel control group	115	Four-week use of Anki flashcard decks covering selected pediatric topics; compared to traditional book and lecture-based study; students were not tracked individually for usage	Anki group improved from pre: 27.93 ± 4.53 to post: 30.80 ± 4.56 (*p* < 0.05) Control group showed no significant change (pre: 27.96 ± 3.70; post: 27.22 ± 5.02) Effect size for Anki group was large (Cohen’s d = 0.8)
Gilbert (2023)	Prospective cohort with non-user control group	130	Participation in an Anki training program; self-reported use vs. no use; usage level stratified by dependency	Anki users (*n* = 78) scored significantly higher than non-users (*n* = 52) across all exams: Course I: +6.4% (*p* < 0.001), Course II: +6.2% (*p* = 0.002), Course III: +7.0% (*p* = 0.002), CBSE: +12.9% (*p* = 0.003)
Haughey (2025)	Mixed-methods prospective	43	Objective tracking of total flashcards reviewed; students grouped into 4 usage levels (0, 1–1,000, 1,001–3,000, > 3,000 cards)	No overall difference in total module score improvement between Anki users and non-users; however, high-use students (> 3,000 cards) showed significantly greater gains than light users and improved physiology performance (*p* = 0.023); students viewed Anki as helpful for retention but time-intensive.
Levy (2023)	Prospective cohort pilot study	45	Tracked usage with a custom Anki Stat Scraper tool; students grouped as heavy, intermediate, light, or limited-Anki users based on flashcard time, cards/day, and number of usage days	No statistically significant difference in anatomy & physiology exam scores across Anki use groups (*p* > 0.05)
Lu (2021)	Cross-sectional survey	201	Self-reported significant use vs. minimal/no use; frequency of full-deck reviews and summer review	Anki users scored higher on USMLE Step 1 than non-users (241.1 ± 13.2 vs. 235.5 ± 17.7; *p* = 0.012) Reviewing past decks “most of the time” or “always” was associated with higher Step 1 scores (246.9 vs. 236.3 for “sometimes”; *p* = 0.010–0.013) Frequent Anki use during summer correlated with higher Step 1 scores (248.7 vs. 238.7–237.3; *p* < 0.001) High-frequency users reported needing to relearn less material during dedicated study (χ² = 38.7, *p* < 0.001) Perceived retention was higher among Anki users than non-users (Wilcoxon *z* = − 7.23, *p* < 0.001)
Magro (2024)	Quasi-experimental study with historical control cohort	206	Faculty-developed Anki flashcards (~ 501 cards) were integrated into pharmacology modules; students were not tracked individually for usage	No statistically significant difference in module pharmacology exams performance between Anki and control cohorts (*p* = 0.43). Students rated flashcards as useful (74%) and reported decreased perceived difficulty with pharmacology topics
Mehta (2023)	Retrospective cross-sectional study	25	Submission of Anki database files logging card count, review frequency, study timing, and duration; usage analyzed via Python and grouped by averaged performance across three NBME-CAS exams	Students with above-median averaged NBME-CAS scores used Anki earlier in the year, reviewed more total flashcards, and used Anki on more days (all *p* < 0.05).
Sun (2021)	Prospective cohort	101	Platform-generated usage data (> 0 flashcard reviews using a pre-generated Anki account); review count and time recorded	No significant association between Anki use and final microbiology/immunology exam scores (*p* = 0.54); 87% of users found Anki helpful, but academic performance was unchanged
Winter (2025)	Cross-sectional correlational study	36	Objective tracking via the Stat Scraper add-on to export quantitative Anki usage data, including total study hours, number of matured cards (interval ≥ 21 days), and number of unsuspended cards.	Mean CBSE = 65.2% ± 11.6 (range 44–86). Students with ≥ 9390 matured cards scored 71.5 ± 10.7 vs. 60.0 ± 9.7 for those below the mean (*p* = 0.002). Total study hours and unsuspended cards were also positively correlated with CBSE scores (*p* = 0.013 and 0.010, respectively). Linear regression identified matured cards as the only independent predictor of CBSE performance (β = 0.649, *p* = 0.026; R² = 0.427).
Wothe (2023)	Cross-sectional survey	161	Daily Anki use vs. non-daily use; self-reported usage analyzed against Step scores	Daily users scored higher on Step 1 (median 238 vs. 233.5; *p* = 0.039); no difference on Step 2 (*p* = 0.440).

### USMLE Step 1/COMLEX Level 1 Performance

Three studies evaluated the impact of Anki usage on USMLE Step 1 performance. Lu et al. [28] included medical students years two through four and found that Anki users scored significantly higher than non-users (241.1 ± 13.2 vs. 235.5 ± 17.7; *p* = 0.012), with even greater improvements among users who frequently reviewed full decks or studied over the summer (up to 248.7 ± 13.3 among consistent users; *p* < 0.001). Users also reported improved retention and reduced need to relearn material during dedicated study (χ² = 38.7, *p* < 0.001). Deng et al. [14] examined students that completed the two-year preclinical curriculum and had taken USMLE Step 1. The authors reported that the number of unique Anki flashcards reviewed during preclinical years independently predicted Step 1 score (β = 5.9 × 10⁻⁴, *p* = 0.024), with each additional ~ 1,700 cards associated with a one-point score increase. Wothe et al. [16] surveyed medical students across all years of training and similarly observed that daily Anki users had higher Step 1 scores than non-daily users (median 238 vs. 233.5; *p* = 0.039). No studies evaluated Anki use in relation to COMLEX Level 1.

### USMLE Step 2/COMLEX Level 2 Performance

Wothe et al. [16] was the only included study to evaluate the relationship between Anki use and Step 2 CK performance. While daily Anki users had slightly higher median Step 2 CK scores than non-daily users (252.5 vs. 247.0), this difference was not statistically significant (*p* = 0.440). Furthermore, Anki use was not an independent predictor of Step 2 CK performance in multivariable models controlling for Step 1 score and class rank. No studies evaluated Anki use in relation to COMLEX Level 2.

### University-Administered Examination Performance

Five studies examined Anki use in the context of university-administered curricula, either through faculty-developed flashcards or objectively logged platform data (e.g., card counts, review time, or activity recorded through Anki or Stat Scraper tools). Results were mixed. In a quasi-experimental study by Durrani et al. [22] fifth-year students assigned to an Anki-based study group showed significant improvement in pediatric topic test scores (mean increase: +2.87 points, *p* < 0.05), with no significant change in the control group and a large effect size (Cohen’s d = 0.8). Gilbert et al. [27] reported that first-year medical students who voluntarily participated in an Anki training program scored significantly higher across three consecutive course exams compared to non-users (Course I: +6.4%, *p* < 0.001; Course II: +6.2%, *p* = 0.002; Course III: +7.0%, *p* = 0.002). In contrast, Levy et al. [25] found no significant difference in anatomy and physiology exam performance among first-year students stratified by Anki usage intensity, despite tracking usage in detail via a custom Anki Stat Scraper tool (*p* > 0.05). Similarly, Sun et al. [23] found no association between Anki use and microbiology/immunology exam scores in a prospective cohort study of first-year students using platform-logged data (*p* = 0.54), though 87% of students reported Anki was helpful. Finally, Magro et al. [15] implemented a pharmacology Anki deck across a course and observed no significant improvement in first-year student exam performance compared to a historical control cohort (*p* = 0.43), though 74% of students rated the tool positively. Finally, Haughey et al. [21] evaluated first-year graduate-entry medical students in Ireland and compared the effectiveness of Anki with traditional study methods such as class notes and third-party question banks. While there were no overall differences in total assessment performance across groups, Anki users demonstrated significantly greater improvement in physiology scores compared with those using other study approaches (+ 35% vs. +6%; *p* = 0.0231). Qualitative feedback further indicated that students perceived Anki as beneficial for consolidating complex material, supporting long-term retention, and enabling flexible, self-paced learning.

### Standardized Preclinical Exams

Three studies evaluated the association between Anki use and performance on institution-administered standardized preclinical exams. Gilbert et al. [27] also reported that Anki users performed significantly better on the CBSE than non-users (+ 12.9%, *p* = 0.003). In a retrospective cross-sectional study, Mehta et al. [26] analyzed Anki usage data submitted by first-year students and correlated it with performance on three National Board of Medical Examiners (NBME) Customized Assessment System (CAS) exams administered at 3-month intervals throughout the first preclinical year. Students with above-median averaged NBME-CAS scores had used Anki earlier in the year, reviewed more total flashcards, and logged a greater number of usage days compared to their peers (all *p* < 0.05). Winter et al. [24] similarly assessed Anki use among 36 preclinical students at the Kirk Kerkorian School of Medicine at UNLV using objective data exported via the Stat Scraper add-on. Higher Anki engagement, measured by total study hours, number of matured cards (interval ≥ 21 days), and number of unsuspended cards, was associated with higher CBSE performance. Students with above-average mature-card counts (≥ 9390) scored 71.5 ± 10.7% compared to 60.0 ± 9.7% for those below the mean (*p* = 0.002). Total study hours and unsuspended card counts were also positively correlated with CBSE scores (*p* = 0.013 and 0.010, respectively). In multivariable linear regression, the number of matured cards was the only independent predictor of CBSE score (β = 0.649, *p* = 0.026; R² = 0.427).

### Patterns of Use and Perceptions

Across studies, the duration and frequency of Anki use ranged from brief interventions to year-long engagement. Most investigations (six of eleven) were cross-sectional and assessed usage at a single time point or within one-course block. Mehta et al. [26] and Winter et al. [24] analyzed longitudinal Anki logs over a single preclinical year, identifying sustained usage patterns with higher total study hours, matured cards, and usage days among higher-performing students. Levy et al. [25] used Stat Scraper data from first-year students during an eight-week anatomy and physiology course to quantify flashcard time and number of active days, while Durrani et al. [22] implemented a structured four-week faculty-developed Anki intervention. Gilbert et al. [27] tracked objective Anki usage from first-year students longitudinally across the first preclinical year following participation in a ten-week Anki training program. Other cohorts characterized usage frequency as daily versus non-daily [16] or regular versus minimal [28]. Haughey et al. [21] collected objective platform data from first-year graduate-entry medical students over an 11-week period to quantify daily, weekly, and total flashcards reviewed, and incorporated qualitative feedback to explore perceived benefits of Anki use. Only three studies assessed students’ subjective perceptions directly: Sun et al. [23] reported that 87% of users found Anki helpful, Magro et al. [15] noted that 74% of students rated the faculty-developed pharmacology deck favorably, and Haughey et al. [21] found that students viewed Anki as beneficial for consolidating complex material, supporting long-term retention, and enabling self-paced learning.

## Discussion

This systematic review identified eleven studies examining the relationship between Anki use and academic performance in undergraduate medical students. Across studies, regular Anki use was associated with modest but consistent improvements in academic performance, particularly on standardized licensing examinations such as the USMLE Step 1 [14, 16, 28]. The magnitude of benefit appeared dose-dependent—students who used Anki more frequently or over longer periods generally achieved higher scores—supporting a cumulative advantage of sustained spaced retrieval. However, because Anki use was not randomly assigned in any included study, this apparent dose–response relationship may partly reflect self-selection, whereby students with greater baseline academic ability, motivation, or available study time are more likely to engage in sustained Anki use. One likely contributor to these benefits is the extended preparation timeline for licensing exams such as Step 1, during which students often engage in several months of cumulative review. This prolonged interval allows for sustained spaced repetition—an ideal condition for Anki’s design. In contrast, only one study [16] assessed Step 2 CK and none evaluated COMLEX outcomes, leaving its utility for more clinically focused or osteopathic licensing exams uncertain.

Findings related to course-based university examinations were more variable. Some studies reported significant gains following faculty-implemented or training-based Anki interventions [22, 27], whereas others using objectively tracked usage found no measurable advantage over traditional study approaches [23, 25]. These inconsistencies likely reflect differences in deck quality, implementation support, and the much shorter preparation timelines typical of in-course exams, where opportunities for distributed review are limited. Nonetheless, several cohorts reported favorable subjective feedback [15, 23], suggesting perceived benefit even when performance differences were not observed.

In contrast, results for standardized preclinical benchmark exams such as the CBSE and NBME assessments were more consistent: students who adopted Anki earlier and used it more regularly tended to perform better [24 [26, 27]. These findings suggest that early, sustained use of Anki supports long-term retention of foundational science content and may be particularly effective when integrated throughout the preclinical curriculum rather than used episodically before exams.

The observed associations between frequent Anki use and stronger performance on standardized exams align closely with established principles of cognitive learning theory. By design, Anki integrates retrieval practice and spaced repetition, both of which have been shown to support long-term retention [4, 6, 29, 30]. The improved Step 1 scores among high-frequency users in multiple studies may reflect not just more time spent, but more effective learning. Several included studies support this interpretation: Mehta et al. [26] found that students who began Anki earlier and reviewed more consistently achieved higher NBME scores, while Deng et al. [14] reported a dose-dependent association between total flashcards reviewed and Step 1 performance. These patterns are consistent with the principle of desirable difficulties, in which introducing appropriate levels of challenge, such as effortful retrieval or problem-solving under increased cognitive demand, enhances long-term memory encoding [4, 9]. The absence of a similar benefit for Step 2 CK in a single study may suggest that Anki is particularly well suited for mastering foundational, fact-based material, but potentially less impactful for assessments emphasizing higher-order clinical reasoning.

Overall, the evidence supports theoretical models that prioritize active, spaced learning over passive review or cramming. Notably, this relationship was more robust for standardized exams than for institution-specific course tests, where findings were more heterogeneous and may have been shaped by variation in deck quality, student engagement, or faculty support. Additionally, the variability in format, content coverage, and grading standards of local examinations may further obscure associations between Anki use and course performance. One possible explanation is the difference in preparation timelines: students typically have several months to prepare for USMLE Step examinations, allowing them to engage in long-term, spaced review. In contrast, university administered exams often follow condensed schedules that may limit the benefits of spaced repetition and reduce opportunities for meaningful Anki use.

The widespread adoption of Anki among medical students, paired with its alignment with evidence-based learning strategies, raises important considerations for educators. Importantly, the findings of this review should not be interpreted as evidence that Anki represents the optimal or sufficient study strategy for all medical students. Rather, Anki appears to be most effective for supporting long-term retention of foundational, fact-based material assessed through standardized examinations. The weaker and more variable associations observed for course-based assessments and Step 2 CK suggest that Anki’s benefits may diminish as assessments place greater emphasis on clinical reasoning, synthesis, and application. Viewed in this context, Anki is best understood as one component of a broader study ecosystem that includes question banks, case-based learning, and integrative review. Overreliance on flashcards may also risk superficial learning if not balanced with deeper conceptual and clinical reasoning [31–33]. Compared with other common board-preparation resources, such as commercial question banks, Anki serves a distinct but complementary function. Question banks have demonstrated strong predictive value for USMLE Step performance, primarily by promoting retrieval practice and test-style reasoning [34, 35], whereas Anki emphasizes distributed reinforcement of factual knowledge. Used together, these approaches may provide a balanced framework for both conceptual understanding and durable retention.

Beyond medical school education, recent evidence supports Anki’s utility across the medical training continuum. In a prospective study of otolaryngology residents, Kuperstock et al. [36] found that structured use of Anki was independently associated with significantly higher in-service exam scores, even after adjusting for prior performance. Similarly, a recent review in radiology education emphasized the growing application of Anki and other spaced repetition tools for improving diagnostic accuracy and memory retention in image-based specialties [37]. Extending beyond residency, a large randomized controlled trial of over 26,000 practicing physicians demonstrated that spaced repetition significantly improved both knowledge retention and transfer in clinical settings [38]. These findings suggest that while evidence for Step 2 CK remains limited, Anki and other spaced-repetition approaches appear to have enduring value across the medical education continuum, extending meaningfully into residency and lifelong learning. As such, educators might consider offering optional training, starter decks, or integration with course objectives while preserving flexibility for individualized study. Supporting the use of spaced repetition may help learners better harness its cognitive advantages.

It is important to distinguish the present findings from prior reviews of Anki use in medical education. A recent narrative review by Mohamed et al. [39] emphasized learner perceptions, quality of life, and attitudes toward Anki, but included only 3 studies that directly evaluated examination performance. In contrast, the present review was conducted using a systematic methodology and synthesized 11 studies specifically examining academic outcomes in undergraduate medical students. This difference in scope is consequential, as positive perceptions or self-reported productivity do not necessarily translate into measurable performance gains. By focusing explicitly on exam outcomes, this review provides a more granular assessment of the magnitude, consistency, and limitations of Anki’s association with academic performance, while also clarifying that observed benefits are context-dependent rather than universal.

### Limitations

This review has several important limitations. First, the majority of included studies were observational or quasi-experimental, and none employed randomized designs. Several relied on historical or convenience control groups, introducing risk of selection bias and limiting causal inference. Second, there was considerable heterogeneity in how Anki use was defined and measured across studies. While some tracked objective usage metrics, others relied on self-report or did not monitor engagement at all. These discrepancies reduce comparability and limit interpretation of findings.

Confounding and self-selection bias are important considerations across the included studies, as students were not randomly assigned to Anki use. High-frequency Anki users may differ systematically from non-users in motivation, baseline academic ability, time spent studying, or use of complementary resources such as commercial question banks. These factors may independently predict examination performance and may contribute to observed associations between Anki use and academic outcomes. Although several studies adjusted for baseline academic metrics such as class rank or prior examination scores, residual confounding cannot be excluded, and causality cannot be inferred.

Additionally, few studies specified what study methods were used by students who did not use Anki, limiting the ability to determine whether observed effects are unique to Anki or reflect broader engagement in retrieval-based learning strategies such as question banks or other flashcard tools. Future research should compare Anki directly with alternative retrieval-practice platforms to clarify whether its algorithmic spacing provides an independent advantage. Third, the duration and context of Anki use varied widely, ranging from structured, faculty-led modules to open-ended, student-initiated usage, which may affect generalizability to other cohorts or curricula. Further, differences in deck quality and content alignment may have contributed to outcome variability. The most widely used AnKing deck is optimized for USMLE-style content and frequently updated by a large student community [10]. In contrast, several studies evaluated institution-specific or faculty-developed decks, which may have varied in flashcard design or structure. Differences in card formatting or construction compared to the widely adopted AnKing deck may have contributed to more variable outcomes. Null results in some studies may therefore reflect suboptimal implementation rather than the ineffectiveness of Anki as a tool. Finally, most studies focused on short-term academic outcomes such as exam scores; few explored long-term retention, clinical reasoning, or downstream impacts on clerkship or licensing performance. Furthermore, several studies evaluated USMLE Step 1 performance as a continuous outcome prior to its transition to pass/fail scoring in 2022. This change limits the ongoing utility of Step 1 scores as comparators and may reduce the relevance of prior findings to current and future cohorts of medical students. Future research should examine whether Anki use influences newer markers of academic and clinical success, such as Step 2 CK and COMLEX performance, clerkship evaluations, and readiness for residency.

## Conclusion

This systematic review found that Anki use among medical students is associated with higher performance on several standardized examinations, including the USMLE Step 1, CBSE, and NBME assessments, findings that are consistent with established principles of spaced repetition and retrieval-based learning that support long-term retention. Evidence for university-administered course examinations was more heterogeneous, likely reflecting variation in deck quality, curricular alignment, and the limited time available for distributed review in course-based settings. Importantly, the observed associations likely reflect the effectiveness of retrieval-based, spaced learning strategies rather than any inherent superiority of the Anki platform itself. As the existing evidence base is predominantly observational, these findings should not be interpreted as causal, nor as evidence that Anki represents an optimal or sufficient study strategy in isolation. Rather, Anki may serve as a useful adjunct within a broader study ecosystem that includes question banks, conceptual integration, and applied learning. Future studies using longitudinal or randomized designs and objective usage tracking are needed to further clarify how spaced-repetition tools contribute to academic performance and long-term knowledge application.

## Supplementary Information

Supplementary Table 1. Methodological quality assessment using the Medical Education Research Study Quality Instrument (MERSQI). Maximum possible score = 18. Item 1: Study design (0–3 points); Item 2: Number of institutions (0.5–1.5 points); Item 3: Response rate (0.5–1.5 points); Item 4: Type of data (1 or 3 points); Item 5: Internal structure validity (0 or 1 point); Item 6: Content validity (0 or 1 point); Item 7: Relationship to other variables validity (0 or 1 point); Item 8: Appropriateness of statistical analysis (0 or 1 point); Item 9: Complexity of statistical analysis (1 or 2 points); Item 10: Outcomes (1, 1.5, 2, or 3 points).
