# Supplementary material for: Anki Use and Academic Performance in Medical Education: A Systematic Review of Evidence and Learning Theory
Source: Med Sci Educ. 2026 Jan 17;36(2):1015–25. doi: 10.1007/s40670-026-02643-5 (PMC13197492; doi:10.1007/s40670-026-02643-5)
Supplement: Supplementary file 1 — Supplementary Table 1. Methodological quality assessment using the Medical Education Research Study Quality Instrument (MERSQI). Maximum possible score = 18. Item 1: Study design (0–3 points); Item 2: Number of institutions (0.5–1.5 points); Item 3: Response rate (0.5–1.5 points); Item 4: Type of data (1 or 3 points); Item 5: Internal structure validity (0 or 1 point); Item 6: Content validity (0 or 1 point); Item 7: Relationship to other variables validity (0 or 1 point); Item 8: Appropriateness of statistical analysis (0 or 1 point); Item 9: Complexity of statistical analysis (1 or 2 points); Item 10: Outcomes (1, 1.5, 2, or 3 points). [file 40670_2026_2643_MOESM1_ESM.docx]

| **Study** | **Item 1** | **Item 2** | **Item 3** | **Item 4** | **Item 5** | **Item 6** | **Item 7** | **Item 8** | **Item 9** | **Item 10** | **Total**  **Score** |
| --- | --- | --- | --- | --- | --- | --- | --- | --- | --- | --- | --- |
| **Deng (2015)** | 1 | 0.5 | 1 | 3 | 0 | 0 | 0 | 1 | 2 | 1.5 | 10 |
| **Durrani (2024)** | 2 | 0.5 | 1.5 | 3 | 0 | 0 | 0 | 1 | 2 | 1.5 | 11.5 |
| **Gilbert (2023)** | 1 | 0.5 | 1.5 | 3 | 0 | 0 | 0 | 1 | 2 | 1.5 | 10.5 |
| **Haughey (2025)** | 1 | 0.5 | 1 | 3 | 0 | 0 | 0 | 1 | 2 | 1.5 | 10 |
| **Levy (2023)** | 1 | 0.5 | 1.5 | 3 | 0 | 0 | 0 | 1 | 2 | 1.5 | 10.5 |
| **Lu (2021)** | 1 | 0.5 | 0.5 | 3 | 0 | 0 | 0 | 1 | 2 | 1.5 | 9.5 |
| **Magro (2024)** | 2 | 0.5 | 0.5 | 1 | 0 | 0 | 0 | 1 | 2 | 1 | 8 |
| **Mehta (2023)** | 2 | 0.5 | 1 | 3 | 0 | 0 | 0 | 1 | 2 | 1.5 | 11 |
| **Sun (2021)** | 1 | 0.5 | 1.5 | 3 | 0 | 0 | 0 | 1 | 2 | 1.5 | 10.5 |
| **Winter (2025)** | 1 | 0.5 | 1.5 | 3 | 0 | 0 | 0 | 1 | 2 | 1.5 | 10.5 |
| **Wothe (2023)** | 1 | 0.5 | 0.5 | 3 | 0 | 0 | 0 | 1 | 2 | 1.5 | 9.5 |

**Supplementary Table 1. Methodological quality assessment using the Medical Education Research Study Quality Instrument (MERSQI).** Maximum possible score = 18. Item 1: Study design (0–3 points); Item 2: Number of institutions (0.5–1.5 points); Item 3: Response rate (0.5–1.5 points); Item 4: Type of data (1 or 3 points); Item 5: Internal structure validity (0 or 1 point); Item 6: Content validity (0 or 1 point); Item 7: Relationship to other variables validity (0 or 1 point); Item 8: Appropriateness of statistical analysis (0 or 1 point); Item 9: Complexity of statistical analysis (1 or 2 points); Item 10: Outcomes (1, 1.5, 2, or 3 points).
